# Sarcopenia, frailty and cachexia patients detected in a multisystem electronic health record database

**DOI:** 10.1186/s12891-020-03522-9

**Published:** 2020-07-31

**Authors:** Ranjani N. Moorthi, Ziyue Liu, Sarah A. El-Azab, Lauren R. Lembcke, Matthew R. Miller, Andrea A. Broyles, Erik A. Imel

**Affiliations:** 1 Department of Medicine, Indiana University School of Medicine, 1120 West Michigan Street CL 365, Indianapolis, Indiana 46202-5111 USA; 2 Department of Biostatistics, Indiana University School of Public Health, Indianapolis, Indiana 46202 USA; 3 Center for Biomedical Informatics, Data Core Services, Regenstrief Institute, Indianapolis, Indiana 46202 USA

## Abstract

**Background:**

Sarcopenia, cachexia and frailty have overlapping features and clinical consequences, but often go unrecognized. The objective was to detect patients described by clinicians as having sarcopenia, cachexia or frailty within electronic health records (EHR) and compare clinical variables between cases and matched controls.

**Methods:**

We conducted a case-control study using retrospective data from the Indiana Network for Patient Care multi-health system database from 2016 to 2017. The computable phenotype combined ICD codes for sarcopenia, cachexia and frailty, with clinical note text terms for sarcopenia, cachexia and frailty detected using natural language processing. Cases with these codes or text terms were matched to controls without these codes or text terms matched on birth year, sex and race. Two physicians reviewed EHR for all cases and a subset of controls. Comorbidity codes, laboratory values, and other coded clinical variables were compared between groups using Wilcoxon matched-pair sign-rank test for continuous variables and conditional logistic regression for binary variables.

**Results:**

Cohorts of 9594 cases and 9594 matched controls were generated. Cases were 59% female, 69% white, and a median (1st, 3rd quartiles) age 74.9 (62.2, 84.8) years. Most cases were detected by text terms without ICD codes *n* = 8285 (86.4%). All cases detected by ICD codes (total *n* = 1309) also had supportive text terms. Overall 1496 (15.6%) had concurrent terms or codes for two or more of the three conditions (sarcopenia, cachexia or frailty). Of text term occurrence, 97% were used positively for sarcopenia, 90% for cachexia, and 95% for frailty. The remaining occurrences were negative uses of the terms or applied to someone other than the patient. Cases had lower body mass index, albumin and prealbumin, and significantly higher odds ratios for diabetes, hypertension, cardiovascular and peripheral vascular diseases, chronic kidney disease, liver disease, malignancy, osteoporosis and fractures (all *p* < 0.05). Cases were more likely to be prescribed appetite stimulants and caloric supplements.

**Conclusions:**

Patients detected with a computable phenotype for sarcopenia, cachexia and frailty differed from controls in several important clinical variables. Potential uses include detection among clinical cohorts for targeting recruitment for research and interventions.

## Background

Skeletal muscle weakness and poor physical performance develop with aging, complicating many chronic clinical conditions and influencing outcomes and decisions regarding the modality and aggressiveness of treatments. Terms used to describe this overall skeletal muscle decline include sarcopenia, cachexia and frailty. The terms used in clinical practice are influenced by the medical subspecialty and the location of medical care [1], though these terms may be used interchangeably despite unique mechanisms and operational definitions [2]. Sarcopenia is a condition of generalized low muscle mass and strength, resulting in poor physical performance complicating aging and many chronic diseases [3, 4]. Cachexia involves catabolism and is tied to nutritional status with resulting extreme weight loss [2]. Most patients with cachexia will have loss of muscle mass and strength consistent with sarcopenia, while patients with sarcopenia may not have cachexia, such as those with sarcopenic obesity [5]. Frailty is the result of aggregate deficits impairing overall functional reserve [6, 7], leading to falls, functional dependence, hospitalizations and other adverse outcomes. Although also tied to musculoskeletal function, frailty is a heterogenous syndrome involving multiple factors including balance, neuropathy, cognitive function, joint dysfunction, cardiovascular function, comorbidities, psychosocial and other factors [8]. Thus, these concepts of frailty, sarcopenia and cachexia are interrelated. Those with cachexia develop sarcopenia; sarcopenia decreases mobility resulting in frailty; and the frail state exacerbates muscular declines [5, 9]. In addition sarcopenia, cachexia and frailty each contribute to functional dependence, dysmobility, disability, hospitalizations, high healthcare costs and death [10–17].

In 2000, disability due to sarcopenia cost the US healthcare system an estimated 18.5 billion dollars [18]. With increasing life expectancy, the public health costs of disability are expected to increase. Studies suggest supervised exercise, dietary supplements and pharmacologic interventions may benefit individuals with sarcopenia [19, 20], frailty [21] and cachexia [22]. However, it is critically important from individual and public health perspectives to identify patients with sarcopenia, frailty and cachexia early for intervention. However these conditions are often not recognized due to lack of knowledge among providers and of equipment for objective measures (e.g. grip strength), as well as time pressures in clinical encounters [23].

Large electronic health record (EHR) datasets combining clinical text notes with coded data provide an opportunity to identify patients having specific conditions from clinical encounters. Natural language processing of text using computers can enhance capture of information by accessing unstructured data from the robust clinical note repository making up the majority of the data within EHR. A computable phenotype is a clinical condition, characteristic, or set of features that can be determined using a computer algorithm to assess its presence or absence solely from data in EHRs and ancillary data sources. Computable phenotypes may include structured data (diagnosis codes, recorded measurements, laboratory values, and medications), unstructured data (text fields or notes), or combinations of such variables.

We hypothesized that patients diagnosed or described by providers in the clinical record as having sarcopenia, cachexia or frailty could be detected using an EHR based computable phenotype combining coded and text data. As evidence of detecting a clinically important phenotype, we hypothesized patients identified based on our computable phenotype would differ regarding clinical features from randomly selected matched controls.

## Methods

### Study design

**Fig. 1:**
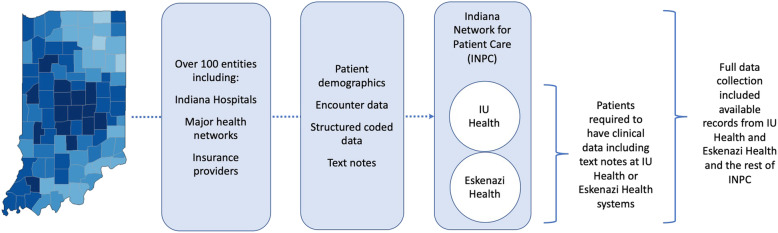
Electronic health record data sources. The Indiana Network for Patient Care (INPC) incorporates data from over 100 health care entities across the state of Indiana. Individual patients may receive care through multiple systems during the study period. To be eligible for this study, patients were required to have clinical data including text notes from the largest contributors to the INPC, IU Health and Eskenazi Health. However, full data collection included available records from these institutions and the INPC as a whole

This was a retrospective case-control study performed using the Indiana Network for Patient Care (INPC), a large statewide clinical data exchange warehouse including over 100 separate healthcare entities including major hospitals, health networks, and insurance providers (Fig. 1). The INPC contains data on over 18 million patients in the form of 7 billion clinical data elements, 1.1 billion encounter records, over 290 million mineable text reports, and data on drug prescription and dispensing. Approximately two thirds of Indiana’s population contribute data to INPC during clinical encounters. This study was conducted in accordance with the Declaration of Helsinki, and prior to the study the protocol was approved by the Indiana University Institutional Review Board. Patients were not contacted during this retrospective study in a large EHR database, and the Indiana University Institutional Review Board approved waiver of consent.

We generated a computable phenotype based on the combination of ICD codes for sarcopenia, cachexia and frailty, and text variants of the words sarcopenia, cachexia and frailty. Both ICD9 and 10 codes for frailty (797, R54) and cachexia (799.4, R64) were assessed. Sarcopenia only has an ICD10 code (M62.84), introduced in 2016. Notes were searched for text terms using locally generated natural language processing software, nDepth. Text searches included detection of variants such as misspellings and grammatical variants. Software also assessed term negation (such as “not sarcopenic”).

### Eligible patients

Included adult patients (18 years of age and older) having encounters and clinical notes within the Indiana University Health System and Eskenazi Health Systems during 2016–2017. The computable phenotyping algorithm was applied to these patients’ records including additional INPC participating institutions during the study period. Patients having one or more positive occurrences during the study period of either the text terms or codes were considered as cases having the computable phenotype. Controls were chosen from the portion of the population assessed that had no occurrences of either the ICD codes or text terms for sarcopenia, cachexia or frailty, matched 1:1 to cases on year of birth (to control for age), sex (male, female) and on race (black, white, other), as recorded in INPC. The index date was the earliest occurrence within the study period of the computable phenotype for cases, or the earliest encounter within the study period for controls.

### Manual validation of computable phenotype

Two clinician investigators reviewed the EHR text around the detected occurrences to confirm whether the computable phenotype algorithm was detecting that the author of the clinical note was attributing the condition to the patient as “present” or “absent”. The two clinicians manually validated all cases detected by the computable phenotype and assigned a value of positive occurrence indicating the condition is present in the patient, negative occurrence indicating the patient does not have the condition (e.g. “not frail” or the description refers to someone else: “she is caring for her sick, frail mother”) or uncertain. Occurrences were rated as positive if both reviewers rated as positive, or if one rated as positive and the other as uncertain. Occurrences were rated as negative if both reviewers rated as negative or if one rated as negative and the other as uncertain. Occurrences were rated as uncertain if both reviewers rated as uncertain or if one reviewer rated as positive and the other as negative. For feasibility, only a smaller subset of 50 randomly selected patients from among the matched controls were manually reviewed. Because controls were based on absence of the computable phenotype terms or codes, manual review of the controls’ entire clinical text notes during the study period was necessary rather than just the text notes near the occurrence of terms or codes as in cases.

### Variables

We extracted additional structured data from INPC on cases and controls, including demographics (used for matching and cohort description), height, weight, body mass index (BMI), diagnosis codes for comorbidities, laboratory values (albumin, prealbumin, and hemoglobin A1C using the closest value to the index date) and hospitalizations. Charlson comorbidity index was calculated to quantify a patient’s overall disease burden [24]. We also assessed records for dispensing of glucocorticoids, dronabinol, megestrol, testosterone and caloric formula supplements (protein shakes, etc.) through Surescripts. Formalized assessments of muscle strength, muscle mass, gait, function, etc. were not accessible and thus could not be detected or analyzed. In EHR data it is not generally possible to distinguish whether absence of a datapoint indicates missing versus absent data.

### Statistical analysis

Continuous variables were summarized by median (1st quartile, 3rd quartile), and categorical variables were summarized by frequency (percentage). For the comparisons between the cases and the controls (total cases and subgroups of cases from the diagnostic categories of sarcopenia, cachexia or frailty), Wilcoxon matched-pair sign-rank test was used for continuous variables, and conditional logistic regression was used for binary variables. For comparisons between cases with and without ICD codes, Wilcoxon rank sum test was used for continuous variables, and Pearson’s chi-square test or Fisher’s exact test was used for categorical variables as appropriate. Two-sided *p*-values < 0.05 were considered statistically significant.

## Results

The computable phenotype detected 10,288 presumptive cases from 2016 to 2017. After manual review, 9594 (93.3%) were considered confirmed positive cases of a clinician identifying the patient as sarcopenic, cachectic or frail. The remaining 694 (6.7%) involved text term use indicating negation or referring to its presence in a separate person (e.g. relative). Most cases were detected by text terms without ICD codes *n* = 8285 (86.4%). All cases detected by ICD codes (total *n* = 1309 (13.6%); sarcopenia *n* = 10, cachexia *n* = 1011, frailty *n* = 329, more than one code *n* = 41) also had supportive text terms. All text term occurrences were manually reviewed as described in the methods, for whether the occurrence indicated a statement regarding presence or absence for the condition. When present, sarcopenia terms indicated presence of the condition 97% of the time (310/318), cachexia terms 90% of the time (3921/4364), and frailty terms 95% of the time (6821/7144). The rest of the occurrences described absence of the conditions. A subset of 50 out of 9594 matched controls were manually reviewed. None had evidence for missed detection of the terms or codes for sarcopenia, cachexia or frailty, and none had other terms sufficient to determine the presence of these phenotypes.

Table 1A is a cross-tabulation indicating the number of cases with each of the individual terms/codes for sarcopenia, cachexia and frailty among the cases. Patients having either the appropriate text term or the ICD code were considered as having the medical condition (i.e. sarcopenia, cachexia and frailty). Patients with an individual text term (or code) for one of the three conditions also frequently had text terms (or codes) for the other conditions. Overall 1496 (15.6%) cases had terms or codes for two or more of the three conditions (sarcopenia, cachexia or frailty) concurrently in their record (*n* = 133 had all three conditions; sarcopenia plus cachexia *n* = 33; sarcopenia plus frailty *n* = 57; cachexia plus frailty *n* = 1273).

**Table 1:** Presence of individual ICD and text terms among the cases (Total *N* = 9594)

**A. Cross-tabulation of ICD codes and text terms.** The diagonal shaded cells indicate the total number of patients with each individual ICD code or text term, while the off-diagonal (unshaded cells) indicate presence of combinations of individual text terms or codes according to row and column of the cell. The numbers in parentheses indicate the percent out of the total N of 9594.						
**n (% of Total N)**	**Text sarcopenia**	**Text cachexia**	**Text frailty**	**Code sarcopenia**	**Code cachexia**	**Code frailty**
**Text sarcopenia**	310 (3.2)	160 (1.7)	188 (2.0)	8 (0.1)	41 (0.4)	14 (0.2)
**Text cachexia**		3921 (40.1)	1239 (12.9)	6 (0.1)	886 (9.2)	108 (1.1)
**Text frailty**			6821 (71.1)	3 (0.0)	451 (4.7)	285 (3.0)
**Code sarcopenia**				10 (0.1)	2 (0.0)	1 (0.0)
**Code cachexia**					1011 (10.5)	38 (0.4)
**Code frailty**						329 (3.4)
**B. Additional information on the presence of ICD codes and text terms.**						
	**Any text term**	**All three text terms**	**No text term**	**Any ICD code**	**All three ICD codes**	**No ICD Codes**
**n (%)**	9594 (100)	129 (1.3)	0 (0)	1309 (13.6)	0 (0)	8285 (86.4)

The median (1st, 3rd quartiles) age of cases was 74.9 (62.2, 84.8) years, with 59% being female. Most were white (69%), 10% black and 21% listed other race. Cases with sarcopenia, cachexia or frailty differed from controls in several clinical aspects (Table 2). The BMI was lower in cases [median 22.1 (18.9, 26.2) kg/m^2^] than controls [28.1 (24.6, 32.5) kg/m^2^]. Cases also had lower serum albumin (*p* < 0.0001) and prealbumin (*p* = 0.0215). Cases had a high odds ratio for diabetes with or without complications compared to controls. Similarly, cases had high odds ratio for hypertension, cardiovascular and peripheral vascular diseases, chronic kidney diseases, liver disease, malignancy, depression, AIDS and neurologic conditions (all *p* < 0.0001). Regarding skeletal health, cases had OR > 3 for osteoporosis and fractures (p < 0.0001). Cases also had higher Charlson comorbidity index scores and were more frequently hospitalized. Cases had more prescribing of dronabinol, megestrol, and nutritional caloric supplements (protein shakes, etc.) but less prescriptions for glucocorticoids and testosterone.

**Table 2:** Clinical variables for cases and controls. Continuous variables are listed as median (1st, 3rd quartiles) with p value by Wilcoxon signed rank test. Categorical variables are listed as n (%) with odds ratio, its 95% CI and *p* value by conditional logistic regression

	Cases	Controls	Odds Ratio (95% CI)	***P*** value
**n**	9594	9594		
**Age**	74.9 (62.2, 84.8)	74.3 (61.6, 84.2)		
**Female**	5663 (59%)	5663 (59%)		
**Race**				
**African American**	981 (10%)	981 (10%)		
**White**	6599 (69%)	6599 (69%)		
**Other**	2014 (21%)	2014 (21%)		
**BMI, kg/m**^**2**^**(*n*** **= 7952 cases and 4897 controls)**	22.1 (18.9, 26.2)	28.1 (24.6, 32.5)		< 0.0001
**Weight, kg (*n*** **= 8638 cases and 6006 controls)**	60.7 (50.5, 73.8)	79.5 (65.9, 94.8)		< 0.0001
**Albumin, mg/dl (*n*** **= 8686 cases and 5409 controls)**	3.6 (3.1, 4.0)	4.1 (3.8, 4.3)		< 0.0001
**Prealbumin, mg/dl (*n*** **= 1336 cases and 105 controls)**	14 (9, 20)	20 (12, 25)		0.0215
**Diabetes with complication**	1247 (13)	337 (3.5)	4.33 (3.80, 4.94)	< 0.0001
**Diabetes without complications**	2412 (25.1)	1547 (16.1)	1.80 (1.67, 1.94)	< 0.0001
**HgbA1C, % (*n*** **= 3379 cases and 1624 controls)**	5.9 (5.5, 6.7)	6.0 (5.6, 6.7)		0.9205
**Hypertension**	6009 (62.6)	3621 (37.7)	3.26 (3.04, 3.49)	< 0.0001
**Cardiovascular disease**	6769 (70.6)	3862 (40.3)	4.49 (4.17, 4.85)	< 0.0001
**Peripheral vascular disease**	1809 (18.9)	590 (6.1)	3.76 (3.38, 4.17)	< 0.0001
**Chronic kidney disease stages 3–5**	2142 (22.3)	690 (7.2)	3.96 (3.59, 4.38)	< 0.0001
**Chronic kidney disease stage 4**	636 (6.6)	151 (1.6)	4.51 (3.75, 5.43)	< 0.0001
**Chronic kidney disease stage 5 or end stage kidney disease**	462 (4.8)	63 (0.7)	8.39 (6.33, 11.12)	< 0.0001
**Any malignancy**	2923 (30.5)	949 (9.9)	4.08 (3.75, 4.45)	< 0.0001
**Liver disease**	1011 (10.5)	252 (2.6)	4.41 (3.82, 5.11)	< 0.0001
**Depression**	1396 (14.6)	173 (1.8)	9.32 (7.86, 11.05)	< 0.0001
**AIDS**	102 (1.1)	15 (0.2)	7.69 (4.32, 13.71)	< 0.0001
**Neurologic conditions**	6205 (64.7)	2061 (21.5)	7.47 (6.87, 8.11)	< 0.0001
**Fractures** (excludes fingers, toes, craniofacial fractures)	1094 (11.4)	321 (3.3)	3.78 (3.21, 4.32)	< 0.0001
**Osteoporosis**	1501 (15.6)	604 (6.3)	3.03 (2.72, 3.37)	< 0.0001
**Charlson comorbidity index value**	3 (1,6)	0 (0, 2)		< 0.0001
**Charlson comorbidity index > 2**	5700 (59)	1519 (16)	8.99 (8.22, 9.85)	< 0.0001
**Number of hospitalizations during 2016 ~ 2017**	2 (0, 4)	0 (0, 0)		< 0.0001
**Selected Medications post-index**				
**Glucocorticoids**	1200 (12.5)	1488 (15.5)	0.77 (0.71, 0.84)	< 0.0001
**Dronabinol**	69 (0.7)	5 (0.1)	13.8 (5.57, 34.21)	< 0.0001
**Megestrol**	93 (1.0)	19 (0.2)	4.90 (2.99, 8.02)	< 0.0001
**Caloric Supplement**	30 (0.3)	12 (0.1)	2.50 (1.28, 4.88)	0.0073
**Testosterone**	14 (0.2)	36 (0.4)	0.39 (0.21, 0.72)	0.0027

Cases having ICD codes for sarcopenia, cachexia or frailty were younger [median (1st, 3rd quartiles) of 68.8 (58.3, 82.1) years] compared to those cases detected by text terms alone (without ICD codes) [75.7 (63.2, 85.1) years] (Supplemental Table 1). Cases with ICD codes also had lower BMI than cases without ICD codes. Diabetes, hypertension, and chronic kidney disease stages 3–5 associated negatively with having ICD codes among cases. However, malignancies, AIDS, osteoporosis, and higher Charlson comorbidity index associated positively with having ICD codes among cases, possibly reflecting greater recognition and priority of coding in these scenarios. Having ICD codes among cases also associated positively with treatments directed at sarcopenia such as use of dronabinol or megestrol.

To determine if the clinical difference from controls was similar in the groups diagnosed with sarcopenia, cachexia or frailty, we conducted a sub-analysis of each group separately and their matched controls, excluding patients with overlapping codes or text terms for more than one of these three conditions. In general, the differences in clinical variables between cases and their matched controls were similar in magnitude and direction when analyzing patient with sarcopenia, cachexia or frailty separately (Supplemental Tables 2a, b and c) as those seen analyzing them together (Table 2). BMI was lowest in the cachexia group. The patients diagnosed with frailty were generally older (median 80.2 years) than those diagnosed with sarcopenia or cachexia (median 64.5 and 63.1 years, respectively) and thus the frailty group had higher proportions of patients with some chronic conditions including diabetes, hypertension, cardiovascular disease, kidney disease, osteoporosis and fractures. However, the proportion of patients with Charlson comorbidity index values over 2 were similar between those with sarcopenia, cachexia or frailty, but in each category was higher than matched controls.

## Discussion

We detected patients having the presence of sarcopenia, cachexia or frailty in the EHR using a computable phenotype incorporating both ICD codes and text terms. Most patients did not have an ICD code to accompany the use of text descriptors. Of note the sarcopenia code was rarely used, accounting for only 10 patients while text terms described sarcopenia in 310 patients in the same time period. This may represent underutilization of the code due to it only being introduced in 2016 [25]. Of the three terms, cachexia was the most likely to be accompanied by an ICD code. However, all terms appeared much more often as text terms than as ICD codes. Clinicians also frequently appeared to use these terms interchangeably, with overlap in their use in 15.6%, occasionally within the same note, suggesting that clinicians may perceive the clinical similarity, and consistent with the research literature surrounding these constructs [5, 9].

Even when the clinicians identify sarcopenia, cachexia or frailty by terms in their notes, these diagnostic codes were only applied 13.6% of the time. The reasons for non-coding may include the clinician’s perceived lesser importance of these medical conditions or a tendency to code only for the primary diseases for which they are seeing the patient. Thus, relying on ICD codes alone for detection in the EHR is insufficient. This finding has clinical relevance because failure to attribute sufficient importance to sarcopenia, cachexia and frailty in the EHR might correspond to failing to target treatment to these conditions. The cases were more likely to have ICD codes if they were male, black, or had malignancy, AIDS, or osteoporosis, or had higher Charlson comorbidity index suggesting greater recognition and coding in these conditions or with greater burden of disease. In a busy clinical practice, sarcopenia, cachexia and frailty may not be diagnosed or coded during physician encounters due to focus on other urgent issues and addressing secondary issues such as skeletal muscle loss may be deferred, delaying detection and treatment.

Cases detected had evidence of systemic disease including more frequent diabetes, hypertension, cardiovascular, peripheral vascular disease, kidney disease, liver disease and malignancy than controls. Cases also had 8.99-fold increased odds of having a Charlson comorbidity index of > 2. Given their larger disease burden, it is not surprising that cases had more hospitalizations during the study period. Similarly, individuals with these conditions of poor muscle health also had higher odds of fractures. In addition, cases had a poorer nutritional status as suggested by lower BMI, albumin and pre-albumin.

Polypharmacy is well documented in frail adults and thought to have a bidirectional effect with temporal associative studies showing that high medication burden may be a cause for frailty, as well as a result [26]. We did not address overall medication burden in this analysis, but instead focused on use of caloric supplement and appetite stimulants, though overall the numbers of patients prescribed these were small. It is possible that caloric supplements (protein shakes, etc.) were not fully captured as these do not require a prescription. The use of megesterol and dronabinol in cases compared to controls is consistent with clinical efforts to manage this cachexia and weight loss [27, 28]. In addition, cases having ICD codes were more likely to receive directed pharmacological treatments. Although testosterone has been used for sarcopenia treatment [29], fewer cases were receiving testosterone than controls. This implies that providers were not prescribing testosterone for this purpose in these sicker patients.

Strengths of our analysis include the large sample size, manual validation of cases and the result that our computable phenotype reliably detected patients that clinicians were diagnosing with sarcopenia, cachexia or frailty. The large sample size with a range of age, gender and race included allows generalizability of results to detect sarcopenia, cachexia or frailty diagnoses within the EHR across a wide range of ages and conditions. Our limitations include that our methods cannot detect sarcopenia, cachexia or frailty if the clinician has not made the diagnosis or documented the codes or the appropriate text in the notes. Additionally, objective assessments for sarcopenia, cachexia or frailty were not performed, therefore the occurrence of codes or terms in EHR does not guarantee that the conditions are present, but only implies that the provider detected or interpreted evidence of these conditions. This results in a potential detection bias and it is likely that our computable phenotype is detecting primarily the sicker patients with these conditions or those with more severe sarcopenia, cachexia or frailty. More mild versions of the clinical phenotypes would thus be missed. Since we are unable to capture scenarios where clinicians failed to detect or mention evidence for sarcopenia, cachexia or frailty, this results in a potentially a large number of missed cases and could introduced a misclassification bias into our analysis if some controls might have clinical features of sarcopenia, cachexia or frailty without documentation. Such misclassification would be likely to decrease the differences between cases and controls for various comparisons. Despite this our groups had significant differences in multiple clinical parameters suggesting that we are truly detecting different groups of patients.

We also found the differences in clinical variables between cases and their matched controls were similar in magnitude and direction when analyzing patients with sarcopenia, cachexia or frailty separately. Overall, our findings, including the considerable overlap in application of these diagnoses, suggest a lack of standardized approach among the general clinicians to both reliably detect these conditions or to differentiate between them. Given that the computable phenotype is dependent on what the clinician labels the patient’s condition, without access to objective measurements we are not able to tell which of the conditions should be most accurately applied to the patient (or if more than one is appropriate). To overcome these biases and limitations, future studies would require further validation of the computable phenotype using objective physical measurements in recruited subjects.

## Conclusions

We validated a computable phenotype to detect diagnosed sarcopenia, cachexia and frailty among patients within EHR. This computable phenotype used the text terms and grammatical variants of the words sarcopenia, cachexia and frailty along with and their associated ICD codes [sarcopenia (M62.84), cachexia (799.4, R64), frailty (797, R54)], which reliably indicated that the clinical provider was labeling the patient as having these conditions. Cases detected in the EHR differed from controls in the frequency of several important comorbidities and number of hospitalizations indicating a clinically meaningful computable phenotype is being detected. Further work is needed to increase electronic capture in the EHR itself of physical measures and components of these physical phenotypes to enable greater detection, differentiation and intervention on a population health level. Such a computable phenotype has wide ranging potential uses clinically in detecting patients at risk for disability, as well as identification for research recruitment for clinical trials.

## Supplementary information

**Additional file 1 Supplemental Table 1.** Comparing cases with text terms only (without ICD codes) versus those with ICD codes.

**Additional file 2 Supplement Table 2.a**: Sarcopenia: Clinical variables for cases defined by sarcopenia text or code or both, and their matched controls. **b**: Cachexia: Clinical variables for cases defined by cachexia text or code or both, and their matched controls. **c**: Frailty: Clinical variables for cases defined by frailty text or code or both, and their matched controls.

**Additional file 3 Supplemental Table 3**. ICD-9 and 10 codes used in the study.

## Data Availability

The datasets generated during the conduct of this study consist of data from individual electronic health records from a health information exchange (the Indiana Network for Patient Care) and cannot be made publicly available due to limitations including contract obligations with the Indiana Health Information Exchange (IHIE), IRB restrictions, and existing data agreements. De-identified versions of these datasets and code can be made available upon request and with appropriate data governance agreements in place.

## Abbreviations

BMI: Body Mass Index
EHR: Electronic Health Record
INPC: Indiana Network for Patient Care
ICD: International Classification of Diseases
