# Supplementary material for: Sarcopenia, frailty and cachexia patients detected in a multisystem electronic health record database
Source: BMC Musculoskelet Disord. 2020 Jul 31;21:508. doi: 10.1186/s12891-020-03522-9 (PMC7395344; doi:10.1186/s12891-020-03522-9)
Supplement: Supplementary file 1 — Additional file 1 Supplemental Table 1. Comparing cases with text terms only (without ICD codes) versus those with ICD codes. [file 12891_2020_3522_MOESM1_ESM.pdf]

Supplemental Table 1. Comparing cases with text terms only (without ICD codes) versus those with ICD codes. Continuous variables are listed as median (1st, 3rd quartiles) with p value by Wilcoxon signed rank test. Categorical variables are listed as n (%) with odds ratio, its 95% CI and p value by conditional logistic regression.

	**Cases with Text terms only (no ICD codes)**	**Cases with ICD codes**	**Odds ratio** (95% CI)	**P value**
**n**	8285	1309		
**Age**	75.7 (63.2, 85.1)	68.8 (58.3, 82.1)		<0.0001
**Female**	4937 (60%)	726 (55%)		0.0048
**Race**				P value <0.0001
**African American**	780 (9%)	201 (15%)		
**White**	5759 (70%)	840 (64%)		
**Other**	1746 (21%)	268 (20%)		
**BMI, kg/m^2 (n= 6868 cases without ICD codes and 1084 cases having ICD codes)**	22.5 (19.4, 26.6)	19.2 (16.8, 22.6)		<0.0001
**Diabetes with complication**	1118 (13.5)	129 (9.9)	1.43 (1.18, 1.73)	0.0003
**Diabetes without complications**	2148 (25.9)	264 (20.2)	1.39 (1.20, 1.60)	<0.0001
**Hypertension**	5158 (63.5)	751 (57.4)	1.29 (1.15, 1.45)	<0.0001
**Cardiovascular disease**	5864 (70.8)	905 (69.1)	1.08 (0.95, 1.23)	0.2259
**Peripheral vascular disease**	1570 (19.0)	239 (18.3)	1.05 (0.90, 1.22)	0.5521
**Chronic kidney disease stages 3-5**	1890 (22.8)	252 (19.3)	1.24 (1.07, 1.44)	0.0040
**Chronic kidney disease stage 4**	570 (6.88)	66 (5.0)	1.39 (1.07, 1.81)	0.0130
**Chronic kidney disease stage 5 or End stage kidney disease**	402 (4.9)	60 (4.6)	1.06 (0.80, 1.40)	0.6733
**Any malignancy**	2487 (30.0)	436 (33.3)	0.86 (0.76, 0.97)	0.0163
**Liver disease**	859 (10.4)	152 (11.6)	0.88 (0.73, 1.06)	0.1732
**Depression**	1189 (14.4)	207 (15.8)	0.89 (0.76. 1.05)	0.1632
**AIDS**	53 (0.6)	49 (3.7)	0.17 (0.11, 0.25)	<0.0001
**Neurologic conditions**	5337 (64.4)	868 (66.3)	0.92 (0.81, 1.04)	0.1831
**Fractures** (excludes fingers, toes, craniofacial)	926 (11.2)	168 (12.8)	0.85 (0.72, 1.02)	0.0796
**Osteoporosis**	1239 (15.0)	262 (20.0)	0.70 (0.61, 0.82)	<0.0001
**Charlson comorbidity index value**	3 (1, 6)	4 (2, 7)		0.0066
**Charlson comorbidity index > 2**	4890 (59.0)	810 (61.9)	0.88 (0.79, 1.00)	0.0505
**Selected Medications post-index**				
**Glucocorticoids**	1013 (12.2)	187 (14.3)	0.84 (0.71, 0.99)	0.0364
**Dronabinol**	47 (0.6)	22 (1.7)	0.33 (0.20, 0.56)	<0.0001
**Megestrol**	72 (0.9)	21 (1.6)	0.54 (0.33, 0.88)	0.0116
**Caloric Supplement**	26 (0.31)	4 (0.31)	1.03 (0.36, 2.95)	0.9604
**Testosterone**	11 (0.1)	3 (0.2)	0.58 (0.16, 2.08)	0.4247
