# Supplementary material for: Sarcopenia, frailty and cachexia patients detected in a multisystem electronic health record database
Source: BMC Musculoskelet Disord. 2020 Jul 31;21:508. doi: 10.1186/s12891-020-03522-9 (PMC7395344; doi:10.1186/s12891-020-03522-9)
Supplement: Supplementary file 2 — Additional file 2 Supplement Table 2.a: Sarcopenia: Clinical variables for cases defined by sarcopenia text or code or both, and their matched controls. b: Cachexia: Clinical variables for cases defined by cachexia text or code or both, and their matched controls. c: Frailty: Clinical variables for cases defined by frailty text or code or both, and their matched controls. [file 12891_2020_3522_MOESM2_ESM.pdf]

**Supplement Table 2.a: Sarcopenia:** Clinical variables for cases and their matched controls. Cases defined by sarcopenia text or code or both, with no text nor code for either cachexia or frailty. Continuous variables are listed as median (1st, 3rd quartiles) with p value by Wilcoxon signed rank test. Categorical variables are listed as n (%) with odds ratio, its 95% CI and p value by conditional logistic regression.

| | **Cases** | **Controls** | **Odds Ratio** (95% CI) | **P value** |
|---|---|---|---|---|
| **n** | 89 | 89 | | |
| **Age** | 64.5 (56.7, 76.9) | 64.0 (55.5, 76.6) | | |
| **Female** | 55 (61.8) | 55 (61.8) | | |
| **Race** | | | | |
| **African American** | 7 (7.9) | 7 (7.9) | | |
| **White** | 52 (58.4) | 52 (58.4) | | |
| **Other** | 30 (33.7) | 30 (33.7) | | |
| **BMI, kg/m$^2$ (n= 71 cases and 46 controls)** | 24.6 (21.2, 28.2) | 29.0 (26.5, 33.9) | | <0.0001 |
| **Weight, kg (n= 74 cases and 56 controls)** | 75.6 (61.0, 88.0) | 87.0 (72.9, 99.3) | | 0.0002 |
| **Albumin, mg/dl (n= 82 cases and 48 controls)** | 3.2 (2.6, 3.8) | 4.2 (3.8, 4.5) | | <0.0001 |
| **Prealbumin, mg/dl (n= 14 cases and 1 controls)** | 13.5 (9.0, 19.0) | 26 **** | | N/A |
| **Diabetes with complication** | 12 (13.5) | 3 (3.4) | 5.5 (1.2, 24.8) | 0.0266 |
| **Diabetes without complications** | 20 (22.5) | 15 (16.9) | 1.5 (0.7, 3.1) | 0.3387 |
| **HgbA1C, % (n= 35 cases and 13 controls)** | 5.6 (3.9, 6.3) | 6.5 (5.8, 7.3) | | 0.5781 |
| **Hypertension** | 44 (49.4) | 23 (25.8) | 3.1 (1.5, 6.3) | 0.0019 |
| **Cardiovascular disease** | 53 (59.6) | 22 (24.7) | 6.2 (2.6, 14.6) | <0.0001 |
| **Peripheral vascular disease** | 14 (15.7) | 4 (4.5) | 6.0 (1.3, 26.8) | 0.0190 |
| **Chronic kidney disease stages 3-5** | 25 (28.1) | 6 (6.7) | 4.8 (1.8, 12.6) | 0.0014 |
| **Chronic kidney disease stage 4** | 9 (10.1) | 3 (3.4) | 3.0 (0.8, 11.1) | 0.0994 |
| **Chronic kidney disease stage 5 or End stage kidney disease** | 5 (5.6) | 1 (1.1) | 5.0 (0.6, 42.8) | 0.1418 |
| **Any malignancy** | 16 (18.0) | 6 (6.7) | 3.0 (1.1, 8.3) | 0.0334 |
| **Liver disease *** | 45 (50.6) | 1 (1.1) | * | <0.0001 |
| **Depression** | 12 (13.5) | 1 (1.1) | 12.0 (1.6, 92.3) | 0.0170 |
| **AIDS **** | 1 (1.1) | 0 (0.0) | ** | ** |
| **Neurologic conditions** | 52 (58.4) | 13 (14.6) | 14.0 (4.3, 45.2) | <0.0001 |
| **Fractures** (excludes fingers, toes, craniofacial fractures) | 7 (7.9) | 2 (2.3) | 3.5 (0.7, 16.8) | 0.1182 |
| **Osteoporosis** | 6 (6.7) | 4 (4.5) | 1.5 (0.4, 5.3) | 0.5299 |
| **Charlson comorbidity index value** | 4 (1, 7) | 0 (0, 2) | | <0.0001 |
| **Charlson comorbidity index > 2** | 57 (64.0) | 11 (12.4) | 24 (5.8, 98.7) | <0.0001 |
| **Number of hospitalizations during 2016~2017** | 2 (0, 4) | 0 (0, 0) | | <0.0001 |
| | | | | |
| **Selected Medications post-index ***** | | | | |
| **Glucocorticoids** | 0 (0.0) | 10 (11.2) | | |
| **Dronabinol** | 2 (2.3) | 0 (0.0) | | |
| **Megestrol** | 0 (0.0) | 0 (0.0) | | |
| **Caloric Supplement** | 1 (0.0) | 0 (0.0) | | |
| **Testosterone** | 0 (0.0) | 1 (1.1) | | |

*: For liver disease, the conditional logistic regression did not converge. The p value was from McNemar test.
**: For AIDS, since the controls had a zero count, no odds ratio can be computed.
***: For medications, conditional logistic regressions did not converge because of zero counts.
****: for pre-albumin, only one control had value (as 26); hence no comparison.

**Supplement Table 2.b: Cachexia:** Clinical variables for cases and their matched controls. Cases defined by cachexia text or code or both, with no text nor code for either sarcopenia or frailty. Continuous variables are listed as median (1st, 3rd quartiles) with p value by Wilcoxon signed rank test. Categorical variables are listed as n (%) with odds ratio, its 95% CI and p value by conditional logistic regression.

| | Cases | Controls | Odds Ratio (95% CI) | P value |
|---|---|---|---|---|
| **n** | 2607 | 2607 | | |
| **Age** | 63.1 (53.4, 74.8) | 62.5 (52.8, 74.2) | | <0.0001 |
| **Female** | 1210 (46.4) | 1210 (46.4) | | |
| **Race** | | | | |
| **African American** | 323 (12.4) | 323 (12.4) | | |
| **White** | 1622 (62.2) | 1622 (62.2) | | |
| **Other** | 662 (25.4) | 662 (25.4) | | |
| **BMI, kg/m2 (n= 2125 cases and 1289 controls)** | 19.8 (17.5, 23.0) | 28.7 (25.2, 33.5) | | <0.0001 |
| **Weight, kg (n= 2343 cases and 1623 controls)** | 56.8 (47.4, 66.9) | 84.5 (71.0, 100.8) | | <0.0001 |
| **Albumin, mg/dl (n= 2385 cases and 1396 controls)** | 3.5 (2.9, 4.0) | 4.1 (3.9, 4.4) | | <0.0001 |
| **Prealbumin, mg/dl (n= 458 cases and 26 controls)** | 12.1 (8.0, 18.8) | 20.5 (9.0, 27.0) | | 0.5000 |
| **Diabetes with complication** | 202 (7.8) | 78 (3.0) | 2.7 (2.1, 3.6) | <0.0001 |
| **Diabetes without complications** | 474 (18.2) | 340 (13.0) | 1.5 (1.3, 1.8) | <0.0001 |
| **HgbA1C, % (n= 773 cases and 416controls)** | 5.8 (5.4, 6.5) | 6.0 (5.6, 6.7) | | 0.8747 |
| **Hypertension** | 1247 (47.8) | 807 (31.0) | 2.3 (2.0, 2.6) | <0.0001 |
| **Cardiovascular disease** | 1532 (58.8) | 822 (31.5) | 3.9 (3.4, 4.5) | <0.0001 |
| **Peripheral vascular disease** | 361 (13.9) | 114 (4.4) | 4.0 (3.2, 5.2) | <0.0001 |
| **Chronic kidney disease stages 3-5** | 342 (13.1) | 120 (4.6) | 3.5 (2.8, 4.5) | <0.0001 |
| **Chronic kidney disease stage 4** | 99 (3.8) | 19 (0.7) | 5.7 (3.4, 9.6) | <0.0001 |
| **Chronic kidney disease stage 5 or End stage kidney disease** | 106 (4.1) | 21 (0.8) | 6.0 (3.6, 10.0) | <0.0001 |
| **Any malignancy** | 1005 (38.6) | 211 (8.1) | 7.8 (6.4, 9.4) | <0.0001 |
| **Liver disease** | 365 (14.0) | 74 (2.8) | 5.7 (4.3, 7.5) | <0.0001 |
| **Depression** | 302 (11.6) | 48 (1.8) | 7.2 (5.2, 10.0) | <0.0001 |
| **AIDS** | 69 (2.7) | 10 (0.4) | 7.6 (3.8, 15.1) | <0.0001 |
| **Neurologic conditions** | 1482 (56.9) | 512 (19.6) | 5.6 (4.9, 6.5) | <0.0001 |
| **Fractures** (excludes fingers, toes, craniofacial fractures) | 201 (7.7) | 55 (2.1) | 4.1 (3.0, 5.6) | <0.0001 |
| **Osteoporosis** | 234 (9.0) | 98 (3.8) | 2.8 (2.1, 3.6) | <0.0001 |
| **Charlson comorbidity index value** | 3 (1, 8) | 0 (0, 1) | | <0.0001 |
| **Charlson comorbidity index > 2** | 1471 (56.4) | 325 (12.4) | 11.2 (9.3, 13.6) | <0.0001 |
| **Number of hospitalizations during 2016~2017** | 2 (0, 4) | 0 (0, 0) | | <0.0001 |
| | | | | |
| **Selected Medications post-index** | | | | |
| **Glucocorticoids** | 263 (10.1) | 373 (14.3) | 0.6 (0.6, 0.8) | <0.0001 |
| **Dronabinol** | 33 (1.3) | 3 (0.1) | 11.0 (3.4, 35.9) | <0.0001 |
| **Megestrol** | 31 (1.2) | 3 (0.1) | 10.3 (3.2, 33.8) | <0.0001 |
| **Caloric Supplement** | 8 (0.3) | 5 (0.2) | 1.6 (0.5, 4.9) | 0.4097 |
| **Testosterone** | 4 (0.2) | 13 (0.5) | 0.3 (0.1, 0.9) | 00393 |

**Supplement Table 2.c: Frailty:** Clinical variables for cases and their matched controls. Cases defined by frailty text or code or both, with no text nor code for either sarcopenia or cachexia. Continuous variables are listed as median (1st, 3rd quartiles) with p value by Wilcoxon signed rank test. Categorical variables are listed as n (%) with odds ratio, its 95% CI and p value by conditional logistic regression.

| | Cases | Controls | Odds Ratio (95% CI) | P value |
|---|---|---|---|---|
| **n** | 5402 | 5402 | | |
| **Age** | 80.2 (69.7, 87.3) | 79.5 (69.1, 86.6) | | |
| **Female** | 3515 (65.1) | 3515 (65.1) | | |
| **Race** | | | | |
| **African American** | 458 (8.5) | 458 (8.5) | | |
| **White** | 3925 (72.7) | 3925 (72.7) | | |
| **Other** | 1019 (18.8) | 1019 (18.8) | | <0.0001 |
| **BMI, kg/m2 (n= 4498 cases and 2803 controls)** | 24.1 (20.9, 28.2) | 27.7 (24.2, 31.9) | | <0.0001 |
| **Weight, kg (n= 4874 cases and 3405 controls)** | 65.5 (54.5, 78.6) | 76.4 (63.6, 90.9) | | <0.0001 |
| **Albumin, mg/dl (n= 4795 cases and 3155 controls)** | 3.7 (3.2, 4.0) | 4.1 (3.8, 4.3) | | <0.0001 |
| **Prealbumin, mg/dl (n= 535 cases and 62 controls)** | 15 (11, 21) | 19 (12, 24) | | 0.2500 |
| **Diabetes with complication** | 863 (16.0) | 203 (3.8) | 5.3 (4.4, 6.2) | <0.0001 |
| **Diabetes without complications** | 1597 (29.6) | 917 (17.0) | 2.1 (1.9, 2.4) | <0.0001 |
| **HgbA1C, % (n= 2069 cases and 928 controls)** | 6.0 (5.5, 6.8) | 6.0 (5.6, 6.7) | | 0.4334 |
| **Hypertension** | 3791 (70.2) | 2218 (41.1) | 3.8 (3.5, 4.2) | <0.0001 |
| **Cardiovascular disease** | 4095 (75.8) | 2392 (44.3) | 4.8 (4.3, 5.3) | <0.0001 |
| **Peripheral vascular disease** | 1108 (20.5) | 380 (7.0) | 3.5 (3.1, 4.0) | <0.0001 |
| **Chronic kidney disease stages 3-5** | 1470 (27.2) | 459 (8.5) | 4.2 (3.7, 4.8) | <0.0001 |
| **Chronic kidney disease stage 4** | 433 (8.0) | 110 (2.0) | 4.2 (3.4, 5.2) | <0.0001 |
| **Chronic kidney disease stage 5 or End stage kidney disease** | 281 (5.2) | 31 (0.6) | 9.9 (6.7, 14.6) | <0.0001 |
| **Any malignancy** | 1412 (26.1) | 591 (10.9) | 2.9 (2.6, 3.2) | <0.0001 |
| **Liver disease** | 399 (7.4) | 132 (2.4) | 3.2 (2.6, 3.9) | <0.0001 |
| **Depression** | 830 (15.4) | 98 (1.8) | 9.8 (7.8, 12.3) | <0.0001 |
| **AIDS** | 15 (0.3) | 5 (0.1) | 3.5 (1.2, 10.6) | 0.0271 |
| **Neurologic conditions** | 3672 (68.0) | 1190 (22.0) | 8.4 (7.5, 9.4) | <0.0001 |
| **Fractures** (excludes fingers, toes, craniofacial fractures) | 692 (12.8) | 225 (4.2) | 3.4 (2.9, 4.0) | <0.0001 |
| **Osteoporosis** | 955 (17.7) | 422 (7.8) | 2.7 (2.4, 3.1) | <0.0001 |
| **Charlson comorbidity index value** | 3 (1, 6) | 0 (0, 2) | | <0.0001 |
| **Charlson comorbidity index > 2** | 3231 (59.8) | 946 (17.5) | 8.0 (7.1, 9.0) | <0.0001 |
| **Number of hospitalizations during 2016~2017** | 2 (0, 3) | 0 (0, 0) | | <0.0001 |
| | | | | |
| **Selected Medications post-index** | | | | |
| **Glucocorticoids** | 705 (13.1) | 872 (16.1) | 0.8 (0.7, 0.9) | <0.0001 |
| **Dronabinol** | 12 (0.2) | 2 (0.0) | 6.0 (1.3, 26.8) | 0.0190 |
| **Megestrol** | 37 (0.7) | 15 (0.3) | 2.5 (1.4, 4.5) | 0.0032 |
| **Caloric Supplement** | 16 (0.3) | 4 (0.1) | 4.0 (1.3, 12.0) | 0.0132 |
| **Testosterone** | 7 (0.1) | 15 (0.3) | 0.5 (0.2, 1.1) | 0.0959 |
