# Supplementary material for: Sarcopenia, frailty and cachexia patients detected in a multisystem electronic health record database
Source: BMC Musculoskelet Disord. 2020 Jul 31;21:508. doi: 10.1186/s12891-020-03522-9 (PMC7395344; doi:10.1186/s12891-020-03522-9)
Supplement: Supplementary file 3 — Additional file 3 Supplemental Table 3. ICD-9 and 10 codes used in the study. [file 12891_2020_3522_MOESM3_ESM.pdf]

Supplemental Table 3: ICD-9 and 10 codes used in the study

Key Condition Codes	***ICD-9***	***ICD-10***
Sarcopenia	No ICD-9 code available	M62.84
Cachexia	799.4	R64
Frailty	797	R54
Osteoporosis and fracture codes		
Fracture codes	733.1-733.16, 733.19, 805-808, 812-813, 818-821, 823, 827-828	M80.00XD, M80.88XA, S02.2XXA, S22.060A-S22.43XG, S32.00A-S32.592A, S42.002A-S42.441D, S52.001D-S52.692A, S62.308D, S62.633A, S72.001A-S72.91XA, S82.102D-S82.92XD, S92.312A-S92.511A
Osteopenia and Osteoporosis	733.0-733.03, 733.09, 733.9, 733.90	M80.x-M81.x
Charlson comorbidity score components		
AIDS/HIV	042.x–044.x	B20.x–B22.x, B24.x
Any Malignancy	140.x–172.x, 174.x–195.8, 200.x–208.x, 238.6	C00.x–C26.x, C30.x–C34.x, C37.x–C41.x, C43.x, C45.x–C58.x, C60.x–C76.x, C81.x–C85.x, C88.x, C90.x–C97.x
Cerebrovascular disease	362.34, 430.x–438.x	G45.x, G46.x, H34.0, I60.x–I69.x
Chronic Pulmonary Disease	290.x, 294.1, 331.2	I27.8, I27.9, J40.x–J47.x, J60.x–J67.x, J68.4, J70.1, J70.3
Congestive Heart Failure	398.91, 402.01, 402.11, 402.91, 404.01, 404.03, 404.11, 404.13, 404.91, 404.93, 425.4–425.9, 428.x	I09.9, I11.0, I13.0, I13.2, I25.5, I42.0, I42.5–I42.9, I43.x, I50.x, P29.0
Dementia	290.x, 294.1, 331.2	F00.x–F03.x, F05.1, G30.x, G31.1
Diabetes (and Complications)	250.0–250.3, 250.8, 250.9, 250.4–250.7	E10.0, E10.1, E10.6, E10.8, E10.9,E11.0, E11.1, E11.6, E11.8, E11.9,E12.0, E12.1, E12.6, E12.8, E12.9,E13.0, E13.1, E13.6, E13.8, E13.9,E14.0, E14.1, E14.6, E14.8, E14.9, E10.2–E10.5, E10.7, E11.2–E11.5,E11.7, E12.2–E12.5, E12.7, E13.2–E13.5, E13.7, E14.2–E14.5, E14.7
Hemiplegia or Paraplegia	334.1, 342.x, 343.x, 344.0–344.6, 344.9	G04.1, G11.4, G80.1, G80.2, G81.x, G82.x, G83.0–G83.4, G83.9
Metastatic Solid Tumor	196.x–199.x	C77.x–C80.x
Mild Liver Disease	070.22, 070.23, 070.32, 070.33,070.44, 070.54, 070.6, 070.9, 570.x, 571.x, 573.3, 573.4,573.8, 573.9, V42.7	B18.x, K70.0–K70.3, K70.9, K71.3–K71.5, K71.7, K73.x, K74.x, K76.0, K76.2–K76.4, K76.8, K76.9, Z94.4
Moderate or Severe Liver Disease	456.0–456.2, 572.2–572.8	I85.0, I85.9, I86.4, I98.2, K70.4, K71.1, K72.1, K72.9, K76.5, K76.6,

		K76.7
Myocardial Infarction	410.x, 412.x	I21.x, I22.x, I25.2
Peptic Ulcer Disease	531.x–534.x	K25.x–K28.x
Peripheral Vascular Disease	093.0, 437.3, 440.x, 441.x,443.1–443.9, 47.1, 557.1,557.9, V43.4	I70.x, I71.x, I73.1, I73.8, I73.9, I77.1, I79.0, I79.2, K55.1, K55.8, K55.9, Z95.8, Z95.9
Renal Disease	403.01, 403.11, 403.91, 404.02,404.03, 404.12, 404.13,404.92, 404.93, 582.x,583.0–583.7, 585.x, 586.x,588.0, V42.0, V45.1, V56.x	I12.0, I13.1, N03.2–N03.7, N05.2–N05.7, N18.x, N19.x, N25.0, Z49.0–Z49.2, Z94.0, Z99.2
Rheumatologic Disease	446.5, 710.0–710.4, 714.0–714.2, 714.8, 725.x	M05.x, M06.x, M31.5, M32.x–M34.x, M35.1, M35.3, M36.0
Additional comorbidities included		
Depression	311	F32.x-F33.x
Hypertension	401-405	I10, I11.x, I13.x, I15.x, I16.x
Neurologic Conditions	294.8, 294.9, 299.01, 299.11, 312.9, 314.01, 331.11, 331.19, 331.81, 352.9, 434.01, 434.11, 435.9, 780.09, 780.93, 784.3, 784.69, 191.6, 191.9, 192.2, 194.3, 225.2, 237.5, 237.70, 270.0, 270.1, 271.0, 272.7, 275.1, 277.5, 279.49, 294.20 299.x, 307.23, 307.81, 311, 317, 318.x, 319, 322.9, 323.9, 324.1, 327.x, 330.x, 334.x, 335.x, 336.x, 339.00, 340, 341.x, 342.90, 343.9, 345.x, 346.x, 347.x, 348.2 348.30, 350.10, 351.0, 351.8, 354.0, 355.9, 356.x, 357.x, 358.x, 359.x, 368.2 368.9, 374.30, 377.x, 386.11, 723.1, 724.2, 728.x, 729.x, 741.90, 759.7, 759.83, 759.89 759.9, 779.0, 780.x, 781.x, 783.42, 784.x, 787.20, 799.59,<br>852.20, 853.00, 854.00, 854.10, V17.89, V18.9, V5883	C71.6, C71.9, C72.0, C75.1, D32.9, D49.6, D89.89, E11.42, E70.0, E70.1, E72.x, E75.x, E76.x, E83.01, F03.90, F04, F09, F32.9, F70, F71, F72, F73, F79, F84.5, F84.8, F90.9, F91.9, F95.2, F95.9, G03.9, G04.90, G04.91, G06.1, G10, G11.x, G12.x, G20, G21.9, G23.1, G24.x, G25.x, G30.9, G31.x, G35, G36.0, G37.9, G40.x, G43.x, G44.x, G45.9, G47.x,<br>G50.0, G51.0, G51.8, G52.9, G56.00, G58.9<br>G60.x, G61.x, G62.89, G63, G70.x-G73.x, G80.9<br>G81.90, G90.9, G93.x, G95.x, H02.409, H46.9, H47.x, H53.x, H81.13, I63.9, M54.x, M60.9, M62.x,<br>M79.x, P90, Q05.9, Q85.00, Q87.x, Q89.x, Q99.2, R13.10, R25.x. R26.9, R27.0, R29.898, R41.x, R47.x,<br>R48.x, R51, R55, R56.x, R62.0, S06.x, Z51.81, Z82.69, Z84.81
Other Cardiovascular Disease	390.x-399.x, 400.x-409.x, 410.x-430.x, 432.x-433.x, 440.x-459.x	I01.x, I02.x, I05.x, I06.x, I07.x, I08.x, I09.x, I11.9, I12.9, I13.x, I15.x, I16.x, I20.x, I23.x-I28.x, I30.x-I39.x, I40.x-I49.x, I51.x-I52.x, I72.x-I75.x, I77.x-I79.x, I80.x, I82.x-189.x, I95.x, I97.x-I98.x
